# Characterization of Recovery in Asphalt Binders

**DOI:** 10.3390/ma13040920

**Published:** 2020-02-19

**Authors:** Fuquan Ma, Xue Luo, Zhiyi Huang, Jinchang Wang

**Affiliations:** College of Civil Engineering and Architecture, Zhejiang University, 866 Yuhangtang Road, Hangzhou 310058, China; mafuquan@zju.edu.cn (F.M.); hzy@zju.edu.cn (Z.H.); wjc501@zju.edu.cn (J.W.)

**Keywords:** asphalt binder, recovery, healing, internal stress, kinetics

## Abstract

The recovery property of asphalt binders plays an important role in the performance and service life of asphalt pavements. Since the internal stress is the driving force for the recovery of asphalt binders, the accurate measurement of the internal stress is full of significance. Based on this rationale, this paper aims to measure the internal stress of asphalt binders using a creep and step-loading recovery (CSR) test and characterizing the recovery behaviors by the internal stress. One base asphalt binder and one styrene–butadiene–styrene (SBS)-modified binder are selected in this study. The key elements of the CSR test are carefully designed and its accuracy is verified in three aspects, including the loading conditions, the effect of disturbance by step-loads, and accuracy of measured internal stress. Then, a kinetics-based recovery model is proposed to evaluate and predict the recovery properties of asphalt binders from its causal relationship. The constant-rate recovery activation energy indicates a major difference with nondestructive and destructive loading conditions, while the fast-rate recovery activation energy keeps almost constant regardless of the loading conditions. After that, the healing activation energy is calculated by using the kinetics-based recovery model and the results indicate that SBS modified asphalt binder shows better healing abilities than a base binder.

## 1. Introduction

Rutting, or permanent deformation is one of the common distresses in asphalt pavements due to the permanent deformation accumulation caused by traffic loads, which has a negative influence on the performance and service life of asphalt pavements. Based on this situation, rutting distress has been a focus for researchers for many years [1,2,3]. There are many factors influencing the resistance to rutting in asphalt mixtures such as binder type, air void content, and bonding stress between aggregate and binder [4]. Among these factors, asphalt binder plays a key role in the performance of asphalt pavements on both the loading and recovery phase, while much less attention is paid to the recovery phase compared with the loading phase, especially for the study of recovery properties in asphalt binders [5].

The recovery potential of asphalt binders is a self-recovery ability of the material, during which the distress level decreases and the performance of asphalt binders enhances with the recovery time. In detail, the deformation of asphalt binders would accumulate continuously under the loading phase, and if the load is destructive then some damaged deformation will generate as well, which cannot be totally restored, even with enough time being available. After loading removal, the accumulated deformation caused by viscoelastic recovery and healing of damaged deformation begins to occur with the increasing of the recovery time. Fully understanding the recovery performance of asphalt binders not only involves understanding its major impact on the performance of the binder itself, but also on the asphalt mixtures and pavements, since the binder plays an important role for them as well. Due to their recovery properties, the asphalt pavements have the ability to “relax themselves” from the continuous traffic loading conditions rather than engaging in unceasing fatigue service. It is a valuable property not only because it involves part of the deformation restoration in this process but also because the service performance is improved a lot with the restoration of deformation. It is also an efficient way to extend the service life of asphalt pavements. In other words, the relaxation ability is fundamental for the enhancement of asphalt mixture design, the improvement of construction method, and the duration performance of the filed asphalt pavements [6]. Thus, it is crucial to study the recovery properties of asphalt binders.

Generally, there are three commonly used experiment methods to study the recovery properties of asphalt binders, including the elastic recovery test [7], the creep recovery test [8,9], and the multiply stress creep recovery (MSCR) test [10,11,12]. These tests were developed for different purposes. The elastic recovery test is mainly used to evaluate the recovery ability of asphalt binders after elasticity deformation. In this test, the asphalt binder sample is pulled into a specified elongation using a ductilometer and then a tool is used to cut into the middle of the sample. The percent of recovery length is determined for all samples to evaluate their recovery properties. The problem of the elastic recovery test is time-consuming and short of accuracy [13]. In order to solve the problem, a creep recovery test is proposed using a dynamic shear rheometer (DSR). The purpose of the creep recovery test is to characterize the recovery properties of asphalt binders after long-time constant loads. An index called a creep recovery compliance is used in this process [9], which is defined as the ratio of recovered strain in the recovery phase to applied stress in the creep phase. Apparently, the creep recovery compliance is not based on the cause-and-effect relationships of recovery, since the recovery response is not driven by the creep stress. The MSCR test is developed based on the creep recovery test, the purpose of which is to simulate the rutting resistance of asphalt binders under slow and heavy traffic loading conditions [14]. This test is also conducted using the DSR and mainly evaluates the recovery properties of asphalt binders at high temperatures. In this process, both the resistance to permanent deformation and the delayed elastic response are measured. A major advantage of the MSCR test is that it can provide lots of information beyond the rutting performance of asphalt binders, and there are no need to run some tests such as elastic recovery and force ductility [15]. Similar to the creep recovery test, the MSCR test is also not based on the cause-and-effect relationships of recovery.

The actual cause of recovery lies in the internal stress, since no external stress is added to the recovery phase. Similar observations have also been reported in metals [16,17] and polymers [18,19]. In 2013, the authors of reference [20] proposed a new method called the creep and step-loading recovery (CSR) test to measure the internal stress in asphalt mixtures, which has gained satisfying results. Considering the fact that the asphalt binder has a significant effect on the recovery of asphalt mixtures, and also studies the recovery properties from its cause-and-effect relationships, the internal stress of asphalt binders must be measured.

This paper aims to measure the internal stress of asphalt binders using the CSR test and characterize the recovery properties of asphalt binders based on the cause-and-effect relationship. The measurement process of internal stress in asphalt binders are introduced in the next section, including the measurement principle of the CSR test and associated procedures. Then, the materials and tests used in this study are presented in Section 3 for the validation of the CSR test method. The CSR tests are conducted at 15 °C, 25 °C, and 35 °C and the author did not conduct the experiments at high temperatures in this study. Section 4 presents verification process of the CSR test in asphalt binders in detail to ensure its accuracy, including the loading conditions, effect of disturbance by step-loads, and the accuracy of measured internal stress. After that, the recovery modulus defined by the internal stress is used to characterize the recovery behaviors of asphalt binders. In addition, a kinetics-based recovery model is proposed to predict the recovery properties in this process. The major conclusions and future work of this study are summarized at the last section.

## 2. Measurement of Internal Stress in Asphalt Binders

According to Teoh et al. [21], there are three commonly used tests to measure the internal stress, including the strain transient dip test, the stress transient dip test, and stress relaxation methods. The CSR test measuring the internal stress of asphalt mixtures is based on the strain transient dip test. Considering that this study aims to propose a similar method to measure the internal stress in asphalt binders, the principles of CSR test on asphalt mixtures are presented next. Then, the details of CSR test for asphalt binders are discussed.

### 2.1. Measurement Principle of the CSR Test

The CSR test is developed by modifying the creep recovery test on asphalt mixtures. During the creep phase, the creep strain increases with the loading time. When the creep load decreases to zero, part of the deformation of the asphalt mixture specimen begins to restore, defined as the recovery strain, which is driven by the internal stress. The remaining deformation is called the residual strain. The difference between the CSR test and creep recovery test is that a continuous step–load is added to the recovery phase to measure the internal stress at one test point, which is indicated by $\sigma_{k}$ (*k* = 1, 2, 3). Thus, both the internal stress and step-loading stress exist in the recovery phase of the CSR test. The difference of the two stresses is called effective stress, which is calculated as:

(1) $$\sigma_{e}=\sigma_{k}-\sigma_{i}$$

where *σ_e_* is the effective stress in the recovery phase, *σ_k_* is the value of step-load, and *σ_i_* is the internal stress. One of the following three situations may happen:

- *σ_k_* < *σ_i_* so *σ_e_* < 0: the residue strain decreases with time, as indicated by $\dot{\varepsilon }<0$.
- *σ_k_* = *σ_i_* so *σ_e_* = 0: the residue strain remains constant, as indicated by $\dot{\varepsilon }=0$.
- *σ_k_* > *σ_i_* so *σ_e_* > 0: the residue strain increases with time, as indicated by $\dot{\varepsilon }>0$.

The principle to measure the internal stress in asphalt mixtures is in accordance with the second situation, as indicated by $\dot{\varepsilon }=0$, in which the internal stress is equal to the step-loading stress. To measure the internal stress at other test points, more step-loads should be added in the recovery phase. Next, a further step is conducted to analyze the possibility of applying the CSR test to measure the internal stress in asphalt binders, which is detailed next.

### 2.2. Design of CSR Test for Asphalt Binders

In previous work, the authors [20] have detailed the key elements in designing the CSR test for asphalt mixtures, including: (1) the duration of the creep load, step-load duration, and ramp time; (2) the number of step-loads and the number of steps in each step-load; (3) the location of every step-load; and (4) the value of every step-load. For asphalt binders, similar rules are followed in designing the CSR test.

For the design of creep load duration, step-load, and loading ramp time, in this study, the creep loading duration time is chosen as 200 s to guarantee that there are enough data to calculate the creep compliance, and to verify the nondestructive and destructive loading conditions using the creep compliance curve, which is detailed in Section 4 “Verification of creep and step-loading recovery test in asphalt binders”. The value of the creep load is adjustable to ensure that the binder is truly nondestructive or destructive. The duration of step-loading time is chosen as 1 s to make sure that few disturbances are added to the recovery of asphalt binder and to determine the strain rate accurately. Different from the CSR test on asphalt mixtures, all of the ramp time is neglected in asphalt binders for the reason that a different instrument is used; the creep load can reach the designed values from zero or decrease to zero from the designed values in an extremely short time using the DSR. Besides, the ramp time cannot be controlled by the DSR.

For the number design of step-loads and number of steps in each step-load, it is different for the nondestructive test and destructive CSR test, since the internal stress is high and diminishes slowly when the asphalt binder sample is damaged. In addition, the number of step-loads and number of steps in each step-load should ensure that there are enough data to gain the change tendency of internal stress under the premise of few disturbances to the recovery of asphalt binders, so both the step-loads and steps in each step-load cannot be too many or too few in number. According to the test experience, it has been proven that six and seven step-loads are appropriate for the nondestructive and destructive CSR tests on asphalt binders, respectively. The number of steps in each step-load is the same as the CSR test on asphalt mixtures, two for undamaged asphalt binders and three for damaged asphalt binders.

For the location design of every step-load, it is chosen based on the characteristic of the internal stress. As indicated in Luo et al. [20], the internal stress is very large at the beginning of the recovery phase and diminishes very quickly within a short period. This means that considerable recovery happens at the beginning of recovery phase and the reduced value gradually becomes stable with the increasing of the recovery time. Thus, more step-loads should be placed at the beginning of recovery period than at the later period. Therefore, the pause between two adjacent step-loads is designed as 1 s, 5 s, 5 s, 10 s, 15 s, and 20 s for undamaged asphalt binders, and 1 s, 6 s, 6 s, 10 s, 10 s, 15 s, and 15 s for damaged asphalt binders. All of the locations of step-loads are proposed based on the test experience.

For the value design of every step-load, it is determined after a number of trial tests. Table 1 gives all of the loading levels of the CSR test in this study based on the testing experience, which can also be regarded as an important reference for the first trail on other asphalt binders. In Table 1, P_N_ and P_D_ is the nondestructive creep load and destructive creep load, respectively. In the nondestructive CSR test, there are six step-loads and each step-load consists of two steps. In the destructive CSR test, there are seven step-loads and each step-load consists of three steps. The values of all these step-loads are shown in this table. For instance, the loading value of step number 1 of step-load number 1 is 20% of creep load P_N_ in the nondestructive CSR test.

**Figure 1:**
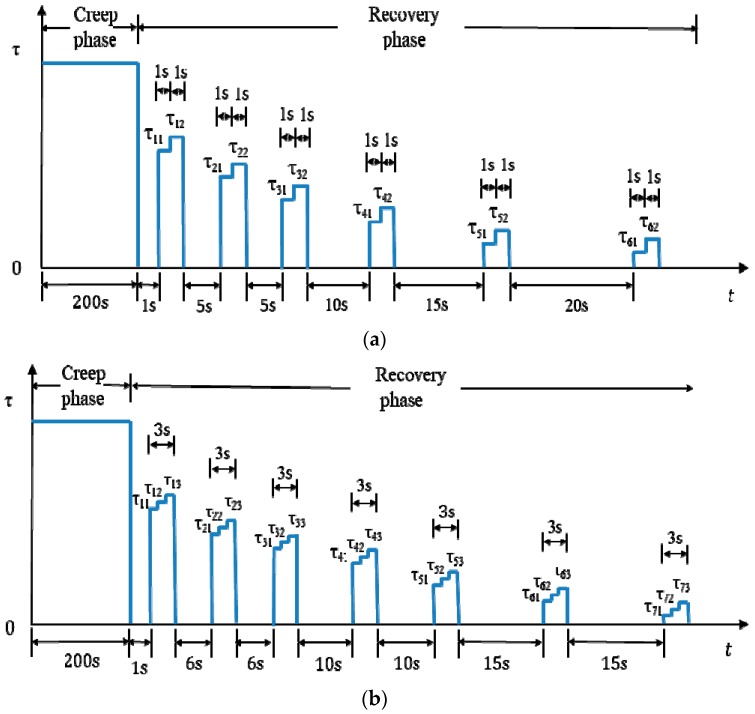
Loading conditions of the creep and step-loading recovery (CSR) test in asphalt binders. (**a**) Nondestructive CSR test; (**b**) Destructive CSR test.

Up to this point, all the necessary design elements of CSR test in asphalt binders have been determined and the detailed information is shown in Figure 1. If the residual strain rate does not accord with zero after the first test, then the value of the step-loads should be adjusted according to the actual situation for next test until the results are acceptable. For example, if $\dot{\varepsilon }<0$ for $\sigma_{21}$ and $\sigma_{22}$, $\dot{\varepsilon }>0$ for $\sigma_{23}$ in Figure 1b, the value of $\sigma_{22}$ will be changed to a lower one in the following tests. Generally, three to five tests are enough to get accurate results for all test points. If the output results are still unacceptable after five tests, a regression method proposed by Teoh [21] should be used to avoid more tests. The purpose of the regression approach is to find a regression model taking $\dot{\varepsilon }$ as Y-axis and the values of step-load as X-axis based on the measured data points, then the intercept of X-axis at which $\dot{\varepsilon }=0$ is regarded as the calculated value of the internal stress. More details can be found in Luo et al. [20].

Up to this point, all of the necessary design elements of the CSR test in asphalt binders have been determined and the detailed information is shown in Figure 1. If the residual strain rate does not accord with zero after the first test, then the value of the step-loads should be adjusted according to the actual situation for next test until the results are acceptable. For example, if $\dot{\varepsilon }<0$ for $\tau_{21}$ and $\tau_{22}$, $\dot{\varepsilon }>0$ for $\tau_{23}$ in Figure 1b, then the value of $\tau_{22}$ will be changed to a lower one in the following tests. Generally, three to five tests are enough to get accurate results for all test points. If the output results are still unacceptable after five tests, then a regression method proposed by Teoh et al. [21] should be used to avoid more tests.

## 3. Materials and Tests

In this study, two kinds of asphalt binder are selected to conduct the mechanical tests, including one base binder and one modified binder with cross-linked styrene–butadiene–styrene (labeled as an SBS modified binder). The two types of asphalt binders are chosen because they are the most commonly used binders in actual pavement engineering projects in southern China, especially in Zhejiang Province. They are all unaged and the basic performance of the two binders is summarized in Table 2 and Table 3.

**Figure 2:**
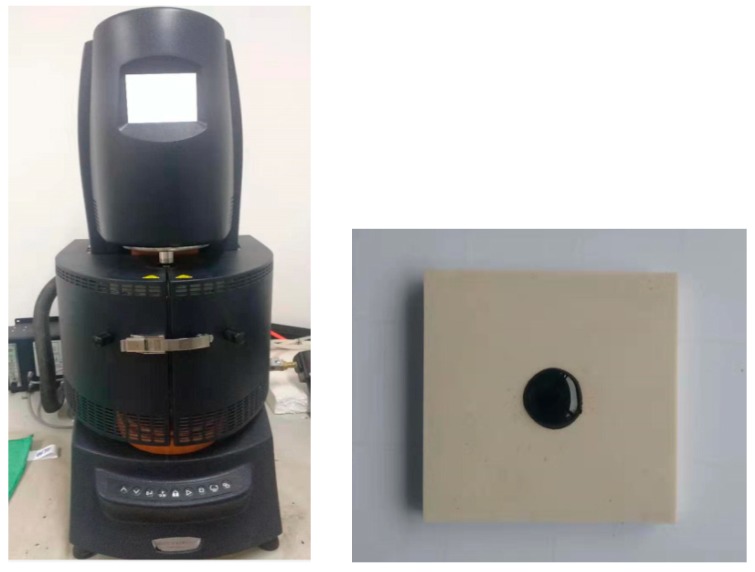
Test equipment and samples.

Three kinds of mechanical tests are conducted in this study, including the nondestructive and destructive CSR test designed above, the creep recovery test, and the creep test, using the DSR manufactured by TA Instruments, USA (model DHR-2). The test equipment and sample used in this study are shown in Figure 2. The parallel plate with a 8-mm diameter and a 2-mm gap value is chosen for the tests. Three test temperatures are conducted for the two asphalt binder samples: at 15 °C, 25 °C, and 35 °C. There are two main reasons for conducting the experiments at these three temperatures. On one hand, the authors referred to the test experience on asphalt mixtures [20] and made some improvements while considering the differences between asphalt mixtures and asphalt binders, especially the temperature sensitivity. On the other hand, the DSR very easily ‘overspeeds’ at high temperatures according to the test experience, even at very low creep loading levels. For each CSR test, the creep load duration and recovery time are all 200 s, so the total time is 400 s. The purpose of conducting the creep recovery test is to measure the residual strain of recovery phase for Section 5 “Characterization of recovery properties for asphalt binders” and to examine whether the effect of disturbance caused by step-loads of CSR test are acceptable for the recovery of asphalt binders. The duration and value of creep load for all samples are the same as that in the CSR test. In addition, the standard creep tests are also conducted with two low loading levels at 15 °C, 25 °C, and 35 °C for both the base binder and SBS modified binder. The creep load duration is also 200 s. The goal of conducting the standard creep test is to verify the nondestructive and destructive loading conditions of CSR tests. More details are shown in the following section.

## 4. Verification of CSR Test in Asphalt Binders

Although the CSR test has been developed and verified for asphalt mixtures, its application for measuring the internal stress in asphalt binders is inventive. Thus, it is necessary to verify the accuracy of the CSR test on asphalt binders. Next, a series of verifications are conducted and the results are discussed, including verification of loading conditions, effect of disturbance by step-loads, and accuracy of measured internal stress.

### 4.1. Verification of Loading Conditions

In order to guarantee that the nondestructive CSR test is truly nondestructive and the destructive CSR test is truly destructive, two more low values of creep load are used to conduct the creep test for all the test samples. The values of the creep compliance of all test samples are examined. Undamaged asphalt binder has similar creep compliance at the same temperature, while a noticeable difference exists if the sample is damaged. The creep compliance is calculated as:

(2) $$D(t)=\frac{\varepsilon_{c}(t)}{\tau_{0}}$$

where D(t) is the creep compliance, $\varepsilon_{c}(t)$ is the creep strain, and $\tau_{0}$ is the creep stress.

**Figure 3:**
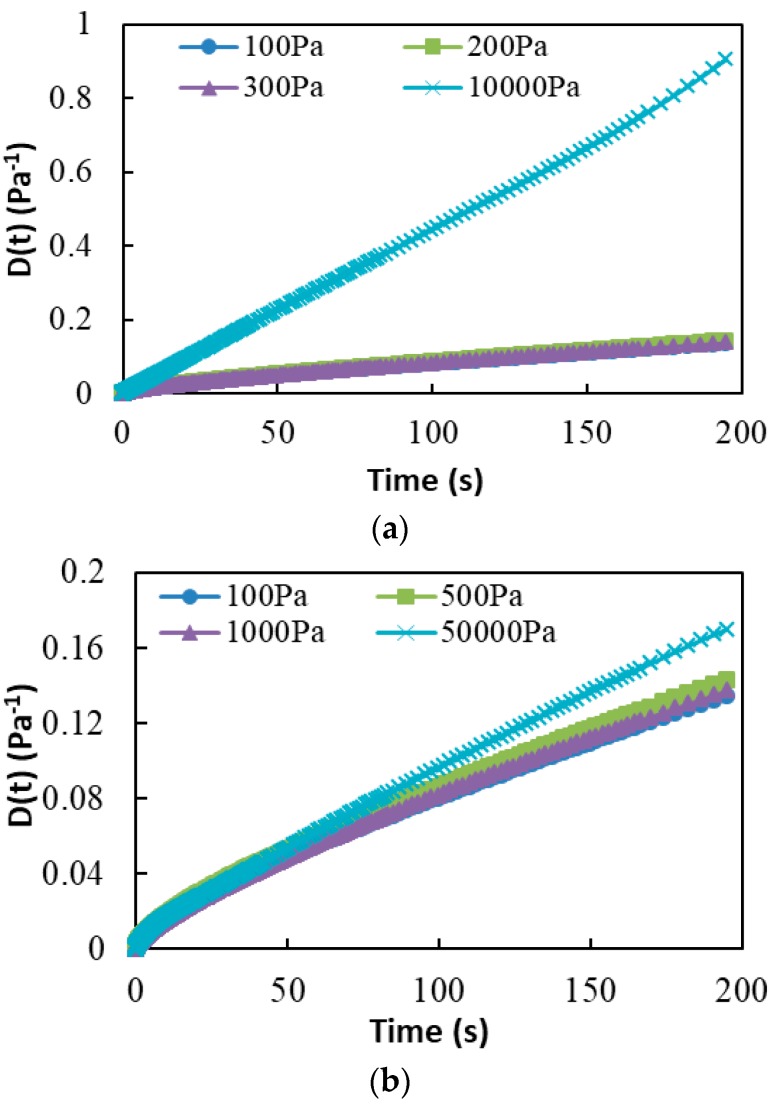
Measured creep compliance of the CSR test. (**a**) Base binder at 35 °C; (**b**) SBS modified binder at 35 °C.

Figure 3 presents the results of D(t) versus the loading time at 35 °C for both the base binder and SBS modified binder. It is obvious that the creep compliance closely matches at the three nondestructive loading levels for both base asphalt binder and SBS modified asphalt binder, while the creep compliance of the destructive loading level indicates significant difference with that at nondestructive loading levels. Therefore, the loading conditions of both nondestructive and destructive CSR tests are verified, while the measured properties of nondestructive and destructive CSR tests are from an undamaged asphalt binder sample and a damaged binder sample, respectively.

### 4.2. Effect of Disturbance by Step-Loads

**Figure 4:**
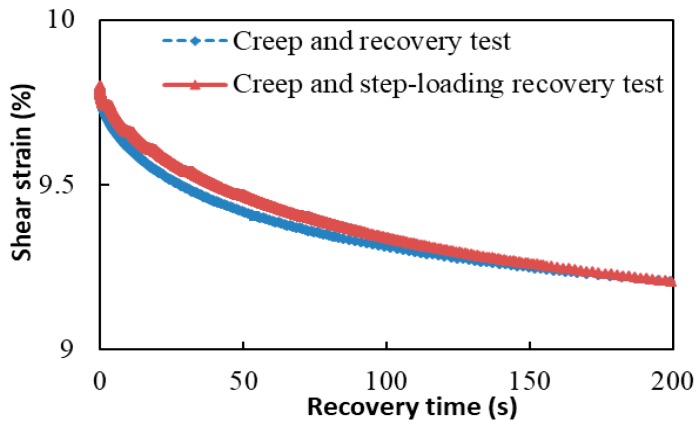
Measured shear strain of the CSR test and creep recovery test.

As mentioned before, the step-loads of the CSR test affect the recovery of asphalt binders. Therefore, the effect of this interruption needs to be verified to see if this influence is acceptable. The verification method is to conduct both the CSR test and creep recovery test on the same asphalt binders under the same creep loading, unloading, and temperature conditions. If the shear strain measured in the CSR test can still match well with that in the creep recovery test after the step-loads, then the disturbance to the recovery of asphalt binders is acceptable. Taking the base binder at 25 °C as an example, shown in Figure 4, it is clear the shear strain measured from the CSR test does not change from that measured from the creep recovery test after the six step-loads. Moreover, the difference between the two test curves is not significant, even under the six step-loads. Therefore, the influence by adding the step-loads to the CSR test is acceptable for the recovery of asphalt binders and the material response measured from CSR test can be regarded as a good way to characterize the recovery properties of asphalt binders.

### 4.3. Accuracy of Measured Internal Stress

**Figure 5:**
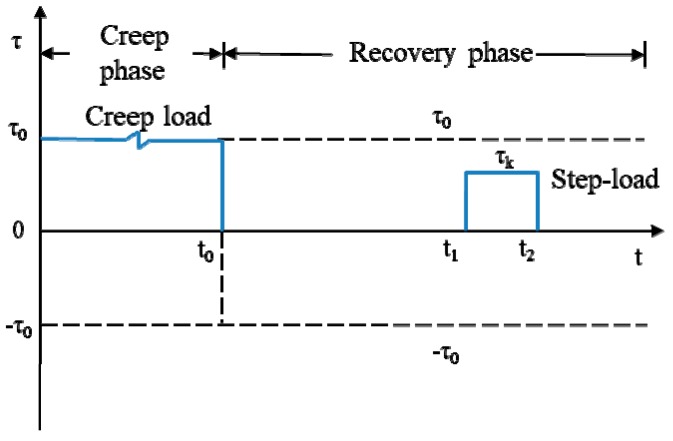
Sketch of calculated internal stress of a nondestructive CSR test.

The internal stress of asphalt binders measured from the CSR test should be checked to ensure that the test is precise and reliable. The verification method involves comparing the measured internal stress with the calculated internal stress of undamaged asphalt binder. If the values match well with each other, then the CSR test is accurate under the undamaged loading conditions. In terms of the Boltzmann superposition principle [27], the residual strain between $\left[t_{1},t_{2}\right]$ can be regarded as the combination results of three stress components, including a positive creep stress $\tau_{0}$, a negative stress $-\tau_{0}$, and a positive creep stress $\tau_{k}$, as shown in Figure 5. For the CSR test, the positive creep stress $\tau_{k}$ represents the measured internal stress at one test point. If $\tau_{21}$ and $\tau_{51}$ in Figure 1a are taken as an example, then if they are both equal to the measured internal stress, $\tau_{k}$ can stand for either of them. The residual strain $\varepsilon_{r}$ between [$t_{1},t_{2}$] can be written as:

(3) $$\varepsilon_{r}=\varepsilon_{r1}+\varepsilon_{r2}+\varepsilon_{r3}$$

where $\varepsilon_{r}$ is the residue strain; $\varepsilon_{r1}$, $\varepsilon_{r2}$, and $\varepsilon_{r3}$ are the residual strain components corresponding to the $\tau_{0}$, $-\tau_{0}$, and $\tau_{k}$, respectively. According to the viscoelastic theory, the residual strain components are calculated as:

(4) $$\varepsilon (t)=\int_{0}^{t}D(t-\xi )\frac{d\tau (\xi )}{d\xi }d\xi \;t\in [t_{1},t_{2}]$$

where D(t) is the creep compliance of the undamaged asphalt binder; and τ(t) is the stress history; ε(t) is the strain corresponding related to the stress; and ξ is the arbitrary time between 0 and t. Substitute $\tau (t)=\tau_{0},t\in [0,t]$;$\tau (t)=-\tau_{0},t\in [t_{0},t]$; and $\tau (t)=\tau_{k},t\in [t_{1},t]$ into Equations (3) and (4), and then take the derivative, which gives the expression of the residue strain rate as:

(5) $${\dot{\varepsilon }}_{r}=\tau_{0}\dot{D}(t)-\tau_{0}\dot{D}(t-t_{0})+\tau_{k}\dot{D}(t-t_{1}).$$

Let ${\dot{\varepsilon }}_{r}=0$, and solve for $\tau_{k}$ from Equation (5), which is calculated as:

(6) $$\tau_{k}=\frac{\tau_{0}\dot{D}(t-t_{0})-\tau_{0}\dot{D}(t)}{\dot{D}(t-t_{1})}.$$

The value of $\tau_{k}$ is the calculated internal stress of undamaged asphalt binder, which can be calculated based on Equation (6) if $\tau_{0}$, D(t), $t_{0}$, and $t_{1}$ are known. It should be noted that D(t) used here must be in the recovery phase, but the existing creep compliance can only be calculated in the loading phase. Thus, a proper model should be used to extrapolate the range of the creep compliance measured in Equation (2). In this study, the power model is used, which is shown as follows:

(7) $$D(t)=at^{b}+c$$

where a, b, and c are fitting coefficients, while t is the creep loading time. Substitute Equation (7) into Equation (6), which gives:

(8) $$\tau_{k}=\frac{\tau_{0}{\left[t-t_{0}\right]}^{b-1}-\tau_{0}t^{b-1}}{{\left(t-t_{1}\right)}^{b-1}}.$$

**Figure 6:**
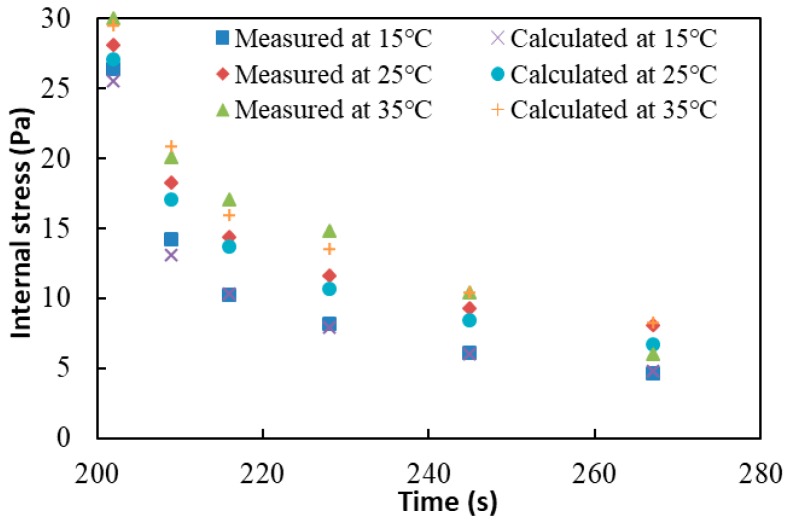
Measured and calculated internal stress of the base binder from a nondestructive CSR test.

As a result, the calculated internal stress of undamaged asphalt binders can be gained via Equation (8). Afterwards, use the base binder as an example. The results of measured internal stresses and calculated internal stresses at 15 °C, 25 °C, and 35 °C are shown in Figure 6. The results demonstrate that the measured and calculated internal stresses match very well with each other, which validates the accuracy of the CSR test. It should be noted that this validation approach is only appropriate for undamaged asphalt binders, considering the fact that only the undamaged binder creep compliance can be calculated using a fitting model in the recovery phase. For the destructive CSR test, the creep compliance cannot be calculated beyond the loading period, so it is of great significance to use the destructive CSR test to measure the internal stress of damaged asphalt binders.

## 5. Characterization of Recovery Properties for Asphalt Binders

After the verification of the CSR for asphalt binders, the next step aims at developing a methodology to evaluate and predict the recovery properties of asphalt binders based on the internal stress. More specifically, two aspects are discussed as follows:

(1) The proposal of the kinetics-based model to evaluate and predict the recovery properties for asphalt binders; and
(2) The analysis of the model results for both undamaged and damaged asphalt binders.

### 5.1. Development of the Kinetics-Based Recovery Model

The kinetics-based model is a mathematical description of changes in properties with respect to time. The purpose is to quantify the rates of a physical or chemical process based on the activation energy. Since Herrington [28] used this model to predict the viscosity of asphalt binders, a lot of researchers have evaluated and predicted the aging properties of asphalt materials based on the kinetics-based model, starting from the carbonyl area [29]. In 2015, the authors [30] developed a kinetics-based aging modulus prediction model in asphalt mixtures. Since then, researchers began to use this model to study the modulus of the asphalt materials [31,32,33]. All of these studies have gained satisfying results, which verifies the accuracy of this approach and also indicates high feasibility of the kinetics-based model. In this section, a further step is taken to explore the feasibility of applying the kinetics-based model to characterize and predict the recovery properties of asphalt binders, which is described below.

Before developing a model to describe the recovery properties of asphalt binders, an appropriate recovery index should be selected first. This study chooses a parameter called the recovery modulus, which was defined by the authors before [20]. It is a material property describing the recovery and healing capacity of asphalt materials after loading removal and the definition of recovery modulus is:

(9) $$G_{r}=\frac{\tau_{i}\left(t\right)}{\varepsilon_{r}\left(t\right)}$$

where $G_{r}$ is the recovery modulus; and $\varepsilon_{r}\left(t\right)$ and $\tau_{i}(t)$ are the residual shear strain and internal stress in the recovery phase of asphalt binders, respectively.

**Figure 7:**
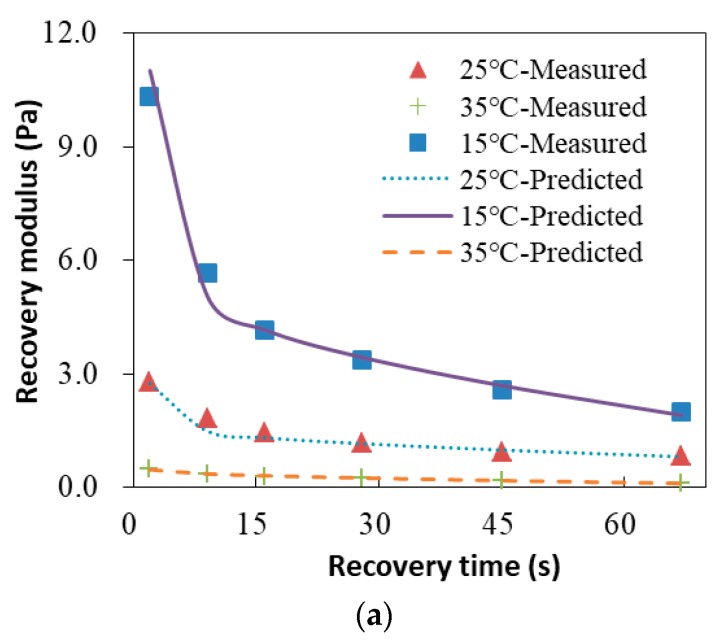

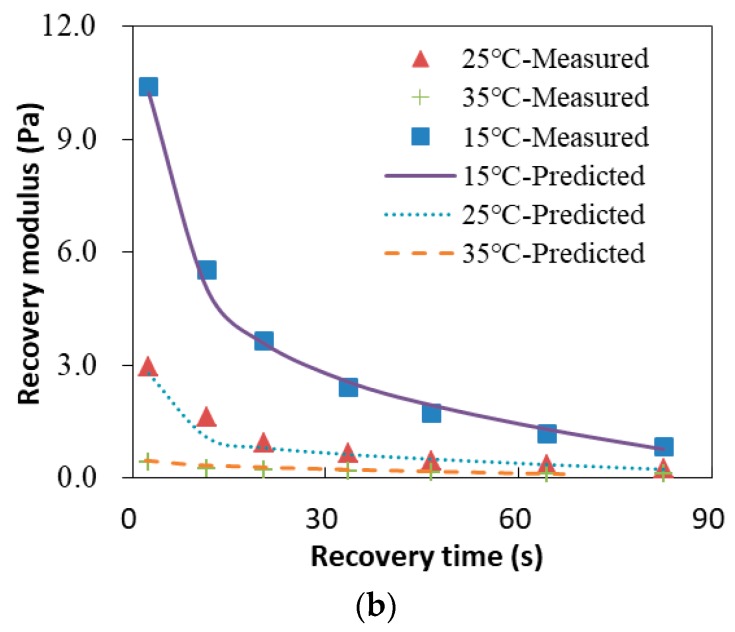
Recovery modulus for the base binder at 15 °C, 25 °C, and 35 °C. (**a**) Nondestructive CSR test; (**b**) Destructive CSR test.

Figure 7 presents the change of the recovery modulus with recovery time for both the undamaged and damaged base binders. For the two kinds of asphalt binders, the recovery modulus decreases with the increase of the recovery time at all test temperatures. Specifically, the recovery modulus decreases rapidly with recovery time at the beginning of the recovery phase and then becomes stable gradually at the end of the recovery period. This reflects that the change of the recovery modulus has two obvious different periods: the fast-rate period and the constant-rate period. Similar results are also observed in the SBS modified binders. Therefore, a model for the prediction of recovery properties of asphalt binders is proposed by integrating the kinetics-based model with Arrhenius equations:

(10) $$G_{r}=G_{ri}+(G_{r0}-G_{ri})(1-e^{-k_{rf}t_{r}})+k_{rc}t_{r}$$

in which

(11) $$k_{rf}=A_{rf}e^{-\frac{E_{arf}}{RT}}$$

(12) $$k_{rc}=A_{rc}e^{\frac{E_{arc}}{RT}}$$

where: $G_{ri}$ is the initial recovery modulus, which is calculated as the ratio of the creep load to the total strain at the end of creep phase; $G_{r0}$ is the intercept of constant-rate line of recovery modulus, which is determined by the measured data points; $t_{r}$ is the recovery time after loading removal; $k_{rf}$ is the fast-rate period reaction constant; $k_{rc}$ is the constant-rate period reaction constant, which is also determined by the measured data points; $E_{arf}$ is the fast-rate recovery activation energy of asphalt binder; $A_{rf}$ is the fast-rate pre-exponential coefficient; $E_{arc}$ is the constant-rate recovery activation energy of asphalt binder; $A_{rc}$ is the constant-rate pre-exponential coefficient; T is the test temperature in Kelvins; and R is the universal gas constant.

There are four parameters which need to be determined in the kinetics-based recovery model: $E_{arc}$, $A_{rc}$, $E_{arf}$, and $A_{rf}$. Among all the parameters, $E_{arc}$ and $A_{rc}$ should be calculated first. Rewrite Equation (12) as:

(13) $$\ln k_{rc}=\frac{E_{arc}}{RT}+\ln A_{rc}.$$

**Figure 8:**
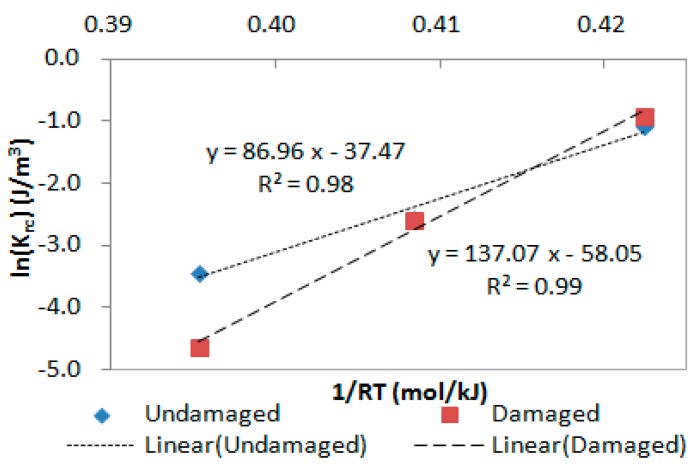
Calculation example of $E_{arc}$ and $A_{rc}$ for base binder at 15 °C, 25 °C, and 35 °C.

Thus, $\ln k_{rc}$ has a linear relationship with 1/RT, and both $E_{arc}$ and $A_{rc}$ can be determined based on Equation (13). An example is given in Figure 8 with the plot of recovery modulus $G_{r}$ versus 1/RT at 15 °C, 25 °C, and 35 °C. It provides a straight line with the slope of $E_{arc}$ and intercept of $\ln A_{rc}$. For instance, the constant-rate recovery activation energy of the undamaged base binder is 86.96 kJ/mol. After that, $E_{arf}$ and $A_{rf}$ are determined using the Excel Solver tool in order to minimize the calculation error.

### 5.2. Analysis of the Modeling Results

The predicted recovery modulus and measured recovery modulus are shown in Figure 7. The results indicate that the predicted values match very well with the measured data at 15 °C, 25 °C, and 35 °C, which verifies the accuracy of the kinetics-based model for recovery of asphalt binders. The results also demonstrate that the recovery modulus decreases with the increasing of temperatures, regardless of whether the nondestructive or destructive CSR test is used.

**Figure 9:**
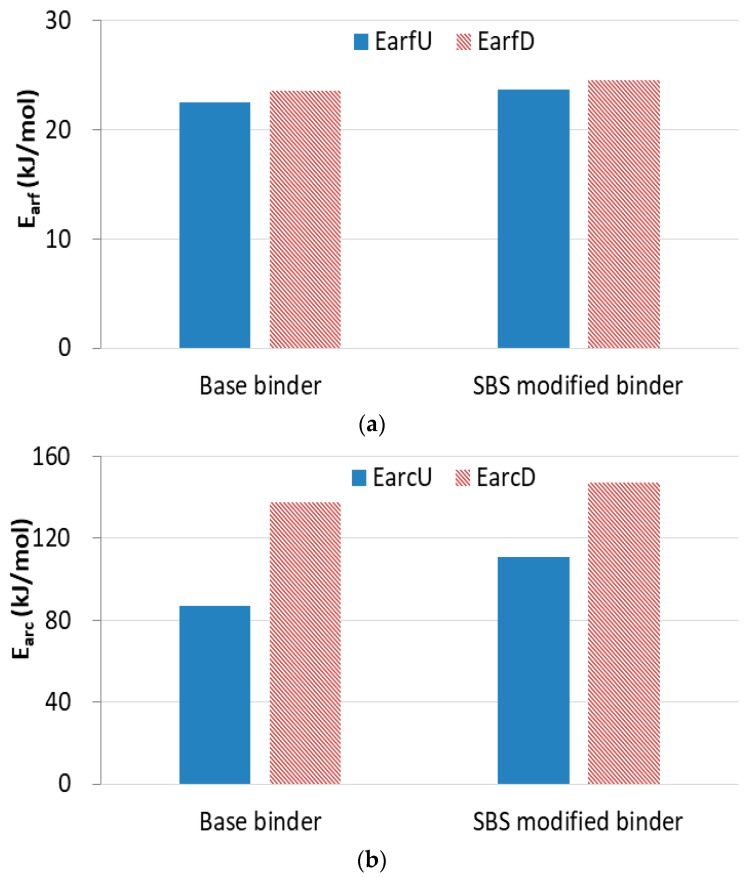
Recovery activation energy of base binder and SBS modified binder. (**a**) Fast-rate recovery activation energy; (**b**) Constant-rate recovery activation energy.

Figure 9 presents results of the fast-rate recovery activation energy ($E_{arfU}$ and $E_{arfD}$) and the constant-rate recovery activation energy ($E_{arcU}$ and $E_{arcD}$) of both base binders and SBS modified binders, where the subscript “U” stands for the undamaged material, and “D” stands for the damaged material. There is viscoelastic recovery plus healing in the damaged material while there is only viscoelastic recovery in the undamaged material, so the constant-rate recovery activation energy increases when the sample is damaged. The results also demonstrate that the fast-rate activation energy does not change a lot, regardless of the loading conditions. This is because the viscoelastic deformation recovers once the load is removed, but the healing of damaged deformation has to wait until the energy is redistributed between the intact material and the crack [34]. In other words, it is relatively easy for the recovery of viscoelastic deformation compared to the healing process. Hence, it is mainly the viscoelastic recovery in the fast-rate period, so the fast-rate activation energy remains almost constant no matter whether the CSR test is destructive or nondestructive.

**Figure 10:**
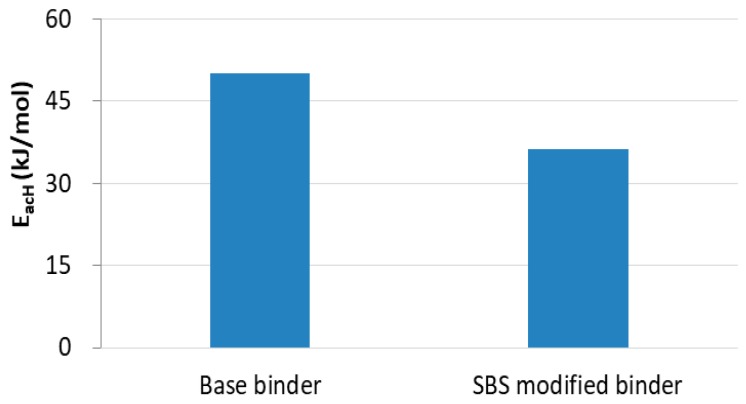
Constant-rate activation energy for healing of base binder and SBS modified binder.

Next, an activation energy-based method is proposed to evaluate the healing abilities of asphalt binders. For an undamaged asphalt binder, only recoverable viscoelastic deformation exists and the deformation would be totally restored if the recovery time duration is long enough. For a damaged asphalt binder, both the recovery of viscoelastic deformation and healing of damaged deformation exist in the material. There is the following relationship for healing based on the constant-rate recovery activation energy [35]:

(14) $$E_{acH}=E_{acD}-E_{acU}$$

where $E_{acH}$ implies the constant-rate healing activation energy and $E_{acU}$ and $E_{acD}$ are the constant-rate recovery activation energy of undamaged and damaged asphalt binders, respectively. The results of constant-rate healing activation energy of the two kinds of asphalt binders are given in Figure 10. It is apparent that the value of $E_{acH}$ of the base binder is higher than that of SBS modified binder, which reveals that the base binder has to overcome greater difficulties than SBS modified binder during the healing process. In other words, SBS modified binder shows better healing abilities than the base binder. Similar observations are also indicated in the literature [36].

## 6. Conclusions and Future Work

This paper measures the internal stress of asphalt binders using the CSR test previously developed for asphalt mixtures by the authors. The accuracy and feasibility of the CSR test on asphalt binders has also been verified. Then, a kinetics-based recovery model is proposed to evaluate and predict the recovery properties of asphalt binders based on the internal stress. The major conclusions can be drawn as follows:

- The CSR test to measure the internal stress in asphalt binders is feasible and accurate at 15 °C, 25 °C, and 35 °C. It is applicable to both the unaged base binder and SBS modified binder.
- For the destructive CSR test, the creep compliance cannot be calculated beyond the loading period, so it is of great significance to use the destructive CSR test to measure the internal stress of damaged asphalt binders.
- The kinetics-based recovery model is developed for both an unaged base binder and SBS modified binder at 15 °C, 25 °C, and 35 °C based on the recovery modulus. The change of recovery modulus with the recovery time can be predicted accurately based on this model.
- The recovery modulus decreases when the temperatures increase from 15 °C to 35 °C. The constant-rate recovery activation energy indicates major differences between nondestructive and destructive loading conditions in unaged asphalt binders, while the fast-rate recovery activation energy stays almost constant regardless of the loading conditions.
- The activation energy for healing is calculated by the constant-rate recovery activation energy of both undamaged and damaged unaged base binder and SBS modified binders. The results indicate that SBS modified unaged asphalt binder has better healing abilities than the unaged base binder.

It is worth mentioning that the CSR tests in asphalt binders of this study have only been conducted and verified on two kinds of unaged asphalt binders at 15 °C, 25 °C, and 35 °C. In a continuation of this work, more types of asphalt binders under higher temperatures and different aging conditions will be developed to further verify the proposed CSR test in asphalt binders.

## Figures and Tables

**Table 1:** Values of the step-load for the first trail.

Step Number	Step-Load Number						
	1	2	3	4	5	6	7
(a) Loading values of the nondestructive test							
1	20%P_N_	10%P_N_	8%P_N_	6%P_N_	4%P_N_	2%P_N_	\
2	40%P_N_	35%P_N_	25%P_N_	15%P_N_	12%P_N_	10%P_N_	\
(b) Loading values of the destructive test							
1	10%P_D_	8%P_D_	6%P_D_	5%P_D_	4%P_D_	2%P_D_	1%P_D_
2	30%P_D_	20%P_D_	15%P_D_	10%P_D_	8%P_D_	6%P_D_	4%P_D_
3	50%P_D_	40%P_D_	30%P_D_	25%P_D_	20%P_D_	15%P_D_	10%P_D_

**Table 2:** Basic performance of the base asphalt binder.

Test Parameters	Requirement	Result	Method
Penetration at 25 °C, 0.1 mm	60–80	74	GB/T 4509 [22]
Softening point, °C	44–57	47.3	GB/T 4507 [23]
Ductility at 5 °C, cm	100(Min)	150	GB/T 4508 [24]
Density at 25 °C, g/cm^3^	—	1.028	GB/T 8928 [25]

**Table 3:** Basic performance of the styrene–butadiene–styrene (SBS) modified asphalt binder.

Test Parameters	Requirement	Result	Method
Penetration at 25 °C, 0.1 mm	40–60	56	T0604-2011 [26]
Softening point, °C	60 (Min)	88.4	T0606-2011 [26]
Ductility at 5 °C, cm	20 (Min)	36.5	T0605-2011 [26]
Density at 25 °C, g/cm^3^	1.01–1.06	1.024	T0605-2011 [26]
